# Free Radicals and Obesity-Related Chronic Inflammation Contrasted by Antioxidants: A New Perspective in Coronary Artery Disease

**DOI:** 10.3390/metabo13060712

**Published:** 2023-05-31

**Authors:** Carlo Caiati, Alessandro Stanca, Mario Erminio Lepera

**Affiliations:** Unit of Cardiovascular Diseases, Department of Interdisciplinary Medicine, University of Bari “Aldo Moro”, 70124 Bari, Italy; alessandrostanca@gmail.com (A.S.); marioerminio.lepera@uniba.it (M.E.L.)

**Keywords:** metabolic syndrome, antioxidants, free radicals, coronary atherosclerosis, oxidized LDL, toxicity

## Abstract

We are surrounded by factors called free radicals (FR), which attach to the molecules our body is made of, first among them the endothelium. Even though FR are to a certain extent a normal factor, nowadays we face an escalating increase in these biologically aggressive molecules. The escalating formation of FR is linked to the increased usage of man-made chemicals for personal care (toothpaste, shampoo, bubble bath, etc.), domestic laundry and dish-washer detergents, and also an ever wider usage of drugs (both prescription and over the counter), especially if they are to be used long-term (years). In addition, tobacco smoking, processed foods, pesticides, various chronic infectious microbes, nutritional deficiencies, lack of sun exposure, and, finally, with a markedly increasing impact, electromagnetic pollution (a terribly destructive factor), can increase the risk of cancer, as well as endothelial dysfunction, owing to the increased production of FR that they cause. All these factors create endothelial damage, but the organism may be able to repair such damage thanks to the intervention of the immune system supported by antioxidants. However, one other factor can perpetuate the state of inflammation, namely obesity and metabolic syndrome with associated hyperinsulinemia. In this review, the role of FR, with a special emphasis on their origin, and of antioxidants, is explored from the perspective of their role in causing atherosclerosis, in particular at the coronary level.

## 1. Introduction

Biogerontologist Denham Harman was the first to discover the concept of free radicals in 1954, while researching an explanation for aging. In his opinion, aging and the degenerative diseases that accompany it were primarily attributable to free radical attacks on cell constituents and connective tissues [1]. Natural antioxidants, on the other hand, are molecules that protect cells against free radicals’ damage; they are critical for maintaining optimum health in both animals and humans [2]. The right balance between the two assures a good equilibrium for the body, shielding from damage to the tissues and organs.

The aim of this review is to shed light on the complex interplay between free radicals and inflammation in the pathogenesis of coronary artery disease, and to discuss the potential of antioxidants used as a therapeutic strategy to counteract their harmful effects.

## 2. The Free Radicals

Free radicals are byproducts of normal cellular metabolism, and can be described as atoms or molecules with one or more unpaired electrons in their valency shell or outer orbit that can exist independently [3]. These unpaired electrons usually give the free radical a high level of reactivity. Since electrons have a strong tendency to exist in a paired rather than an unpaired state, free radicals steal electrons from other atoms indiscriminately, converting these atoms into secondary free radicals, thereby triggering a chain reaction that can cause significant biological damage to all the basic molecules (lipids, proteins, and DNA) that make up living matter. In short, in stealing electrons, they operate as “terrorists” in the body.

The most important class of free radicals produced in living systems is those derived from oxygen [4]. We know that oxygen is one of the most important molecules necessary for life: aerobic organisms cannot exist without it. However, we must recognize that oxygen is at the same time one of the most dangerous poisons for the maintenance of life because of its strong oxidant power. Davies referred to this contradicting element of aerobic existence as the “paradox of aerobic life” [5]. Oxygen free radicals, or reactive oxygen species (ROS), are byproducts of normal cellular metabolism, as are reactive nitrogen species (RNS). ROS and RNS are widely known to act as both toxic and beneficial species, having either a damaging or a useful effect on biological systems [6]. ROS are created from so-called “oxidation” reactions which lead to a kind of “biological rusting”, an effect caused by too much oxygen in biological tissues [7]. Hydroxyl (OH•), superoxide (O_2_•-), nitric oxide (NO•), nitrogen dioxide (NO_2_•), peroxyl (ROO•), and lipid peroxyl (LOO•) are some examples of free radicals. In addition, hydrogen peroxide (H_2_O_2_), ozone (O_3_), singlet oxygen (^1^O_2_), hypochlorous acid (HOCl), nitrous acid (HNO_2_), peroxynitrite (ONOO-), dinitrogen trioxide (N_2_O_3_), and lipid peroxide (LOOH) are not free radicals, but can readily lead to free radical reactions in biological systems [8]. They can all play a significant role in the damage wrought upon nucleic acids, lipids, proteins, and cell structures. The radical hydroxyl, for example, is known to react with all DNA molecule components, disrupting both the purine and pyrimidine bases, and the deoxyribose backbone [9]. This must obviously be considered one of the most important elements that can play a role in the pathogenesis of several diseases. In fact, the permanent alteration of genetic material caused by these “oxidative damage” occurrences is considered, by various authors, to be the initial stage in mutagenesis, cancer, and aging [10]. Moreover, many different forms of protein oxidative alteration can be caused either directly by ROS or indirectly by oxidative stress processes [11]. These can attack enzymes and proteins, disrupting normal cell activities, or cell membranes, producing a chain reaction of destruction. In addition, by attacking free amino groups in proteins, oxidants generate oxidative stress, which leads to the development of advanced lipoxidation and glycation end products (ALEs and AGEs, respectively). We know that changes in cell signaling and functioning brought on by increased AGEs and ALEs formation can result in protein cross-linking and aggregation, which can damage and kill cells [12].

The production of free radicals in the body is continuous and inevitable. Immune system cells, for instance, create free radicals and ROS for use as weapons. In particular, phagocytes exploit microorganisms rapidly releasing free radicals, thanks to the so-called “respiratory burst” [13]. They have the ability to create large quantities of superoxide and hydrogen peroxide, increasing oxygen intake and metabolizing significant amounts of glucose. In this way, both bacteria and viruses can promote oxidative stress in cells during infection, inducing tissue damage, thrombosis, and red blood cell dysfunction. For instance, this has been shown in COVID-19 disease, in which excessive levels of ROS have been associated with a greater severity of the condition, suggesting the possible use of antioxidants to prevent sudden worsening of the clinical situation [14]. Nevertheless, the majority of free radicals are produced in the cells’ mitochondria, which are the body’s primary molecular source of energy and are found in great concentrations in practically every kind of human cell. This energy producing generates adenosine triphosphate (ATP), which is the fundamental molecule serving to store and transfer energy in cells, but that also inevitably generates free radicals as toxic waste. The cell undergoes a number of metabolic processes, each of which can produce different free radicals. Thus, even a single cell can produce many different kinds of free radicals, causing damage to mitochondrial components and starting the degrading process which underlies the so-called “Free Radical Theory of Aging” [15].

## 3. The Pathogenesis of Coronary Artery Disease

Coronary artery disease is still one of the main killers in Western societies [16]. Several approaches have recently been attempted to contrast this disease [17,18], but the results are not reassuring and still preliminary; the real problem is that the only way to cure a disease is by eliminating those factor(s) that provoked it.

**Figure 1:**
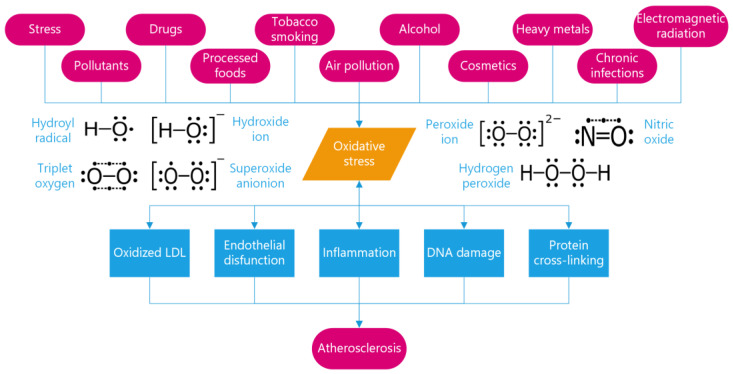
Mechanisms of free-radical-induced pathology in cardiovascular diseases. Free radicals, induced by triggers such as pollution and other toxic substances, can trigger oxidative stress, leading to oxidized low-density lipoprotein (LDL), endothelial dysfunction, inflammation, and DNA and protein damage. These factors can contribute to the development and progression of atherosclerosis and coronary artery disease.

Today, it is universally accepted that free radicals also play a key part in the pathogenesis of atherosclerosis, and so are one of the most important causes of clinical complications, the most significant of which is coronary artery disease. It has been widely demonstrated that large amounts of reactive oxygen are linked to a loss of coronary artery pliability [19]. Free radicals play a role in the entire atherogenic process, from endothelial dysfunction through the rupture of a lipid-rich atherosclerotic plaque, leading to acute myocardial infarction or sudden death (Figure 1) [20]. Since Steinberg and his colleagues initially proposed the low-density lipoprotein (LDL) oxidation theory for atherosclerosis in 1989 [21], significant data have accumulated supporting the concept that oxidative alteration of LDL is the crucial starting event in the genesis of atherosclerosis [22,23]. Cholesterol is surely one of the most essential substances for human life; it is fundamental for producing hormones, vitamin D, and bile acids and cannot be considered alone as the main risk factor for coronary heart disease, as so many clinicians mistakenly think. However, the situation changes when cholesterol becomes the object of attack by free radicals. Many studies have shown that oxidized LDL can cause serious health problems, encouraging endothelial cells to generate inflammatory markers, participating in foam cell production, wreaking cytotoxic effects on endothelial cells, reducing tissue macrophages’ movement, and inhibiting nitric oxide-induced vasodilation [24]. Moreover, it is now known that oxidation of LDL lipids and apolipoprotein B 100 makes LDL pro-atherogenic [25], and that oxidation of HDL inhibits its intrinsic anti-atherogenic capabilities [26]. The resulting membrane damage in the cells that line our blood vessels can lead to hardening and thickening of the arteries and eventually to heart attacks and strokes. Finally, free-radical attacks on collagen can cause cross-linking of protein molecules, stiffening the tissues.

The practical approach to investigating free radicals is to analyze the by-products of free radical pathology, as it is extremely difficult to measure free radicals directly. Free radicals, in fact, exist for only a minuscule amount of time (fractions of seconds) [27]. Some of these by-products of oxidative stress (biomarkers) can be measured and have been proposed and investigated in relation to cardiovascular diseases, such as isoprostanes, malondialdehyde, oxidized LDL, glutathione, and myeloperoxidase [28,29]. Recent studies reported that some oxidative stress biomarkers, such as oxidized LDL, asymmetric dimethylarginine, total thiol, and malondialdehyde-modified LDL were associated with cardiovascular events in patients with stable coronary artery disease [30]. However, none of them have been established as a reliable and independent predictor of cardiovascular events in clinical practice. This may be due to several limitations, such as lack of standardization, variability, confounding factors, and low specificity. Moreover, oxidative stress biomarkers may reflect different aspects of the oxidative process, such as lipid peroxidation, protein oxidation, and antioxidant capacity, and may not capture the overall oxidative balance in the body [29]. Therefore, to establish the clinical utility of oxidative stress biomarkers, more large-scale clinical trials are needed to determine their value in addition to established models of cardiovascular risk prediction. Furthermore, future research should focus on the development of improved biomarkers that can be incorporated into standardized clinical chemistry tests, facilitating their widespread use in clinical practice. Nevertheless, we believe that combining biomarkers of oxidative stress with non-invasive tests of endothelial coronary function, such as the cold pressure test [31,32], which can now be performed with high feasibility using enhanced transthoracic Doppler echocardiography [33,34,35,36], may provide a powerful insight into coronary atherosclerosis. While biomarkers express the level of oxidative stress occurring, endothelial tests investigate the extent to which this oxidative stress penetrates the coronary endothelium, which can vary from individual to individual [31,37]. This combination can be particularly useful in detecting coronary atherosclerosis in its early stages [38] and assessing the vulnerability of plaques in more advanced cases [39].

### 3.1. Factors Causing Excessive Free Radical Production

#### 3.1.1. Stress

The pressures common in industrial societies can trigger stress responses. We all know that stress conditions are widely considered by clinicians as one of the most important causes of the development of various diseases. In fact, due to the increased respiratory oxygen use and metabolic turnover during periods of stress, free oxygen radicals are created in abundance. A high oxygen intake is necessary to fulfill the increased energy demand under stressful situations brought on by unfavorable environmental factors, difficult and heavy work, and psychological trauma [40]. Several studies in animals demonstrated that exposure to external stimuli such as hypoxia, hypothermia, hyperthermia, and immobilization led to an increased formation of reactive oxygen and nitrogen species, causing severe damage to DNA, proteins, and lipids. This could be reduced by the use of antioxidants [41,42,43,44]. Moreover, the hormones that mediate the stress reaction in the body—cortisol and catecholamines—will themselves degenerate into particularly destructive free radicals [45,46]. It has also been demonstrated that myocardial necrosis produced by catecholamines could be attributed to a free-radicals-mediated mechanism, which may lead to a particular form of catecholamine-induced cardiomyopathy [47]. Researchers now know one way stress may cause disease: a stressful life mass-produces free radicals.

#### 3.1.2. Pollutants

The pollutants produced by modern technologies often generate free radicals in the body. The food most of us buy contains farming chemicals, including fertilizers and pesticides, that produce free radicals when we ingest them. Nowadays, the use of this kind of substances is visibly growing because they are still the most effective and economical way to enhance the yield, regardless of its quality. It is widely known that exposure to pesticides is associated with the pathogenesis of several diseases including cancer and cardiovascular diseases. The reason for this is also their ability to increase ROS and oxidative stress production, which cannot be properly counteracted by the intracellular antioxidant system [48]. For instance, studies have shown that glyphosate, an herbicide widely used to control the weeds that compete with crops, can determine an overaccumulation of ROS and a reduction of the NADH and NADPH pool of the cells, compounding its cytotoxic and genotoxic effects [49,50]. Different studies, in fact, demonstrated increased serum biomarkers of oxidative stress in the blood of agricultural workers exposed to pesticides for a long time, together with a remarkable decrease in antioxidant enzyme levels [51,52].

#### 3.1.3. Drugs

Prescription drugs can often have the same effect as pesticides; their harmful side-effects may be caused by the free radicals they generate. Several studies have suggested the possible role of oxidative stress in clinically relevant drug-related side-effects. For example, doxorubicin, one of the most commonly used anthracyclines, has been implicated in the generation of ROS in cardiomyocytes and lipid peroxidation, clearly justifying the well-known cardiac toxicity typical of this class of antineoplastic drugs [53,54]. However, also, other widely prescribed drugs, such as paracetamol and common nonsteroidal anti-inflammatory drugs, have been associated with an increased production of reactive metabolites, a depletion of antioxidants, and the activation of proapoptotic proteins. These mechanisms may underlie their well-known hepatotoxic and nephrotoxic side effects [55,56,57]. Clearly, therefore, prescribing drugs indiscriminately without considering the role of drug-induced oxidative stress in the overall benefit-risk assessment, can be very dangerous.

#### 3.1.4. Processed Foods

Processed foods, especially meat, frequently contain high levels of lipid peroxides, which do not only affect the organoleptic and functional characteristics of the food, but also contribute to producing free radicals and toxic substances that can harm consumers’ health and contribute to the development of diseases [58].

#### 3.1.5. Tobacco Smoking

Cigarette smoking exposes smokers to a complex mixture of carcinogenic and poisonous compounds, as well as stable free radicals and ROS, which can contribute to the significant biological oxidative damage of the cells. There is also a well-documented synergistic effect with environmental respirable particles, such as asbestos fibers, coal dust, and diesel exhaust particles [59]. Tobacco-related free radicals mainly attack the arteries (coronaries in particular) and lungs. The immunologic battle at the level of the plaque exacerbates the situation, since more damage is provoked and the plaque core can enlarge, causing a rapid progression of the narrowing and eventually rupture of the plaque, giving rise to acute events (myocardial infarction and sudden death). Thus, smoking maximally provokes endothelial dysfunction [60], reduces coronary flow reserve [61], increases the progression of coronary artery disease [62], causes coronary spasm [63], and increases mortality [64]. Regarding the lungs, it is now known that much of the lung damage associated with smoking is caused by free radicals, which can overpower the lungs’ antioxidant defenses and activate a number of proapoptotic and proinflammatory signaling pathways, leading to different lung diseases, such as asthma, Chronic Obstructive Pulmonary Disease (COPD), and cancer [65].

#### 3.1.6. Air Pollution

Air pollution has similar effects. In recent years, various researchers have focused on the so-called Environmentally persistent free radicals (EPFRs). EPFRs are a recently discovered class of combustion products that persist in fine particles for a long time. They can generate toxic ROS such as hydroxyl radicals that promote oxidative stress, mediating adverse health effects in respiratory and cardiovascular diseases, including COVID-19 disease [66,67].

#### 3.1.7. Alcohol

Alcohol is a potent generator of free radicals (although red wine contains antioxidants that counteract this effect). Through a variety of processes, largely occurring in the liver, alcohol stimulates the production of ROS and interferes with the body’s natural defensive systems against these chemicals. For example, alcohol breakdown in the liver results in the synthesis of molecules that are then metabolized in the cell, resulting in the generation of ROS. Alcohol also increases the activity of cytochrome P450 enzymes, which contribute to ROS generation. Furthermore, alcohol can affect the amounts of specific metals in the body, promoting the creation of ROS. Finally, alcohol lowers the amounts of antioxidants that can remove ROS [68,69]. All these effects can have a fundamental role in the development of alcoholic liver disease and its progression to liver fibrosis.

#### 3.1.8. Cosmetics and Cleaning Products

Cosmetics and household cleaning products have been linked to increased levels of oxidative stress in the body. This is largely due to the fact that many of these products contain man-made chemicals, such as phthalates, parabens, and triclosan, which are known to generate free radicals and induce oxidative stress [70,71,72]. Furthermore, these chemicals are absorbed through the skin and mucous membranes and bypass the liver, which would normally metabolize and detoxify them before their entry into the bloodstream. Additionally, inhaling these substances can cause oxidative stress and inflammation of the airways, effects that are similar to those observed in individuals suffering from COPD and asthma [73,74]. As a result, cumulative exposure to these chemicals over time can lead to an increased risk of chronic diseases, such as cardiovascular and respiratory disorders and cancer [75,76]. Therefore, it is important to limit exposure to these chemicals by choosing natural and organic personal care products and cleaning agents whenever possible, as well as avoiding any unnecessary and excessive use of these products altogether.

#### 3.1.9. Heavy Metals

Heavy metals, including those used in prosthetic manufacturing, have been recognized as another important source of oxidative stress and inflammation in the body. Among the most common sources of heavy metals are amalgam fillings, which contain about 50% mercury and other metals such as silver, copper, and tin. Mercury is a highly toxic metal that can produce free radicals and reduce antioxidants such as glutathione [77]. Several studies have shown that people with amalgam fillings have higher levels of mercury in their hair, blood, urine, and tissues than those without [78,79,80,81,82]. Moreover, mercury can pass from the mother to the fetus or the infant, causing negative effects to cognitive- and neurodevelopment [83,84]. Mercury can indirectly lead to the development of atherosclerosis by raising the levels of total cholesterol, triglycerides, and LDL-C, while lowering the level of HDL-C. Thus, mercury can be regarded as a risk factor in the progression of atherosclerosis [85,86,87,88,89]. Other dental procedures that involve metal implants such as pins and capsules can also release ions and particles that interact with the surrounding tissues, inducing the expression of pro-inflammatory cytokines and chemokines. These molecules can recruit and activate inflammatory cells such as monocytes, macrophages, T cells, and mast cells to the arterial wall, where they can uptake oxidized LDL, transformed into foam cells. Foam cells are the main component of atherosclerotic plaques, which can grow and rupture, causing thrombosis and ischemia [90,91]. Some studies have described titanium, one of the metals most commonly used in dental implants, as a potential atherosclerosis risk factor [92,93].

#### 3.1.10. Chronic Infections

Moreover, in root canal infections, bacteria can locally produce ROS or induce oxidative stress in host cells, leading to inflammation and necrosis of the pulp tissue, pain and tooth loss, and also causing systemic inflammation and endothelial dysfunction [94]. Local oxidative stress can also facilitate the translocation of bacteria from the oral cavity to the systemic circulation, which can contribute to the development of atherosclerotic plaques and coronary artery disease [95,96,97,98,99]. Studies suggest that eliminating and reducing the presence of periodontal bacteria in subgingival plaque may be a crucial prophylactic measure for preventing both periodontitis and atherosclerosis [100].

#### 3.1.11. Coronary Stents

Coronary stents can also induce oxidative stress and inflammation in the vascular wall, which can lead to adverse outcomes such as restenosis, endothelial dysfunction, and stent thrombosis. A correlation between levels of pro/antioxidant and pro/anti-inflammatory markers and the development of in-stent re-occlusion lesions has been demonstrated. Imbalances in these biomarkers can lead to cross-activation of pro-inflammatory and pro-oxidative stress pathways, further exacerbating the formation of these complex lesions [101]. Oxidative stress and inflammation can modulate the expression and activity of various molecules involved in the vascular remodeling process, such as nicotinamide adenine dinucleotide phosphate oxidase (NOX), nitric oxide synthase (NOS) and proteins regulating mitochondrial function [102,103,104]. Therefore, targeting oxidative stress and inflammation may be a promising strategy to improve the outcome of coronary stent placement.

#### 3.1.12. Electromagnetic Radiation

Lastly, free radicals can result from all types of electromagnetic radiation. Nowadays, technological devices have become indispensable parts of daily life. However, their harmful effects on the body, particularly the neurological system, are widely documented [105,106]. Recent studies did not only show that electromagnetic field exposure produces oxidative stress in diverse tissues, but also that it causes substantial changes in blood antioxidant marker levels, causing symptoms such as fatigue, headache, and cognitive impairment [107]. Some studies have suggested that exposure to radiofrequency electromagnetic waves (RF-EMF) from cell phones may induce oxidative stress, inflammatory response, and hypothalamic-pituitary-adrenal (HPA) axis deregulation, all of which are risk factors for atherosclerosis [108,109]. However, even if the cellular target of RF-EMF is still controversial, some studies have identified the plasma membrane as a possible site of interaction, where it could increase ROS formation by boosting the activity of plasma membrane NADH oxidase [110]. Moreover, the effects of RF-EMF on the genesis of heart tumors, cardiac arrythmias, and myocardial damage have been widely described [111,112,113]. Exposure to sunlight also generates free radicals that age the skin, causing roughness and wrinkles. If the exposure is prolonged, skin cancer may ensue [114].

All the above-cited damaging factors can, through the formation of free radicals, generate endothelial damage and trigger inflammation in the coronary wall. However, the immune system’s normal repair effect (rejuvenation phase) should take place after eliminating the offender and stopping the process. Unfortunately, another factor comes into play at this point, perpetuating the inflammatory state at the coronary level and preventing the inflammation from stopping: metabolic syndrome, another inheritance of our modern society.

## 4. The Major Role of Metabolic Syndrome in Endothelial Dysfunction

Endothelial dysfunction plays a pivotal role in the pathogenesis of cardiovascular diseases and is an early marker of atherosclerosis [115]. Metabolic syndrome is a cluster of metabolic abnormalities including insulin resistance, hyperglycemia, hypertension, central obesity, and dyslipidemia, and is associated with an increased risk of cardiovascular diseases [116]. Insulin resistance and hyperinsulinemia, the hallmark features of metabolic syndrome, are key contributors to endothelial dysfunction by promoting a state of chronic low-grade inflammation [117].

Insulin resistance is caused by obesity, which results from an unbalanced diet rich in industrial refined products with a high glycemic index, combined with a sedentary lifestyle [118]. This promotes the storage of excess calories as fat, along with toxic waste (heavy metals, pesticide, etc.) and inflammatory fats such as arachidonic acid in visceral fat deposits [119]. This storage is not inert, because thanks to lipase activity, the fat moves continuously from the storage back into the blood and to all other organs, first and foremost the endothelium. This process has been referred to as the metastatic spread of toxic fat [120]. When this occurs, full insulin resistance takes place.

Insulin resistance and hyperinsulinemia promote the production of pro-inflammatory cytokines such as tumor necrosis factor-alpha (TNF-α) and interleukin-6 (IL-6), leading to increased oxidative stress and impaired nitric oxide (NO) bioavailability, which in turn promotes endothelial dysfunction [121,122]. Furthermore, insulin resistance is associated with a decreased expression of endothelial nitric oxide synthase (eNOS) and increased expression of inducible nitric oxide synthase (iNOS), leading to a decreased NO production and increased production of ROS [123].

In addition, insulin resistance and hyperinsulinemia have been shown to promote the activation of the nuclear factor kappa-light-chain-enhancer of activated B cells (NF-κB) pathway, which is a key regulator of inflammation [124,125,126]. This pathway boosts the expression of genes involved in the production of pro-inflammatory cytokines, chemokines, adhesion molecules, and enzymes involved in the synthesis of eicosanoids, such as cyclooxygenase-2 and lipoxygenase, which play a role in the perpetuation of the inflammatory response [127].

Moreover, the insulin-induced strong activation of delta-6 and delta-5 desaturase, which are enzymes involved in the metabolic pathway of linoleic acid conversion to arachidonic acid, leads to an increase in the production of several pro-inflammatory eicosanoids including prostaglandins, thromboxanes, and leukotrienes, which contribute to the development of atherosclerosis [128,129,130,131].

Recommendations: To reduce the levels of linoleic acid in the diet, we recommend opting for fat sources that are low in omega-6 fatty acids, such as olive oil or nuts, while reducing the intake of red meat. Lowering insulin levels requires a reduction in the glycemic load of the diet, which can be achieved by increasing the consumption of fruits and vegetables as the primary sources of carbohydrates and reducing the intake of high-glycemic carbohydrates, such as grains and starches. To successfully tackle metabolic syndrome, it is important to consume grains and starches that come from unprocessed and unrefined wheat free of chemicals such as glyphosate. Additionally, the timing of the grinding process cannot be underestimated: wheat ground more than 4 weeks before is totally oxidized and does not contribute to improving health, but rather worsens metabolic syndrome [132]. The more oxidized the wheat, the higher the glycemic index and the greater the oxidative stress [118]. Therefore, wheat should be used soon after grinding and discarded after 4 weeks [132]. Additionally, it is important to limit the intake of saturated fatty acids, as they can activate the inflammatory pathway by indirectly activating NF-κB [133]. Finally, a constant intake of fish oils, which are rich in omega-3 fatty acids, can directly inhibit the formation of arachidonic acid or dilute its concentration in target cells’ membranes, especially in adipose tissue, reducing the overall inflammation in the body [134]. Unfortunately, fish, especially larger species such as tuna, are often contaminated with chemicals, particularly mercury, due to the terrible man-made contamination of the sea and ocean [135]. Therefore, even the consumption of this otherwise healthy food should be limited to only once or twice a week at most. Alternatively, modulation of the inflammatory arachidonic acid can be achieved by consuming nuts and seeds, such as walnuts, which have a high content of linoleic acid, the precursor of all eicosanoids, but also by maintaining low delta-5 desaturase enzyme activity by keeping the insulin levels low in the blood [136].

## 5. Free Radicals Defenses

Given the many sources of free radicals, it is not surprising that all aerobic forms of life maintain elaborate anti-free-radical defense systems, also known as antioxidant systems. Antioxidants are electron donors. They can break the free radical chain reaction by sacrificing their own electrons to feed free radicals, but without turning into free radicals themselves [137]. Some antioxidants are produced by the body, but some are not. In addition, the body’s natural antioxidant production can decline with age [138].

The system is highly complex and not well understood. However, the most likely attack points for the excess free radicals are the essential fatty acids in the cell membrane. This implies the need to neutralize these oxidized lipids and so remove the source of oxidation from the body. This requires three distinct types of antioxidants: fat-soluble, surface-active, and water-soluble. Fat-soluble antioxidants such as vitamin E, coenzyme Q10, and beta-carotene neutralize free radicals in the membrane and become partially stabilized free radicals [139]. Then, they have to be shuttled to the blood stream in order to be eliminated by the liver or by the kidney. To do that, a water-soluble antioxidant such as vitamin C is needed [140]. However, there is another fundamental step in this work of neutralization, which is to carry the free radical from inside the membrane to the blood: this is the job carried out by the surface-active antioxidants [141].

This class of substances is not proteins or vitamins, but comes from the vegetables reign: they are polyphenols. Without them, the body has no chance to get rid of antioxidants and complete the detoxification process.

However, such molecules have very important added properties:

- Repairing damaged molecules—Some unique types of antioxidants can repair damaged molecules by donating a hydrogen atom. This is very important when the molecule is a critical one, such as DNA [142];
- Blocking metal radical production—Some antioxidants have a chelating effect—they can grab toxic metals such as mercury and arsenic, which can cause free radicals’ formation, and “hug” them strongly so as to prevent any chemical reaction from taking place. Water-soluble chelating agents can also escort toxic metals out of the body through the urine [143];
- Stimulating gene expression and endogenous antioxidant production—Some antioxidants can stimulate the body’s genes and increase the natural defenses [144];
- Providing a “shield effect”—Antioxidants, such as flavonoids, can act as a virtual shield by attaching to DNA to protect it from free radicals’ attacks [145];
- Provoking cancer cells to “commit suicide”—Some antioxidants can provide anti-cancer chemicals that halt cancer growth and force some cancer cells to self-destruct (apoptosis) [146].

## 6. Chemical Structure and Biological Functions of Dietary Polyphenols

**Figure 2:**
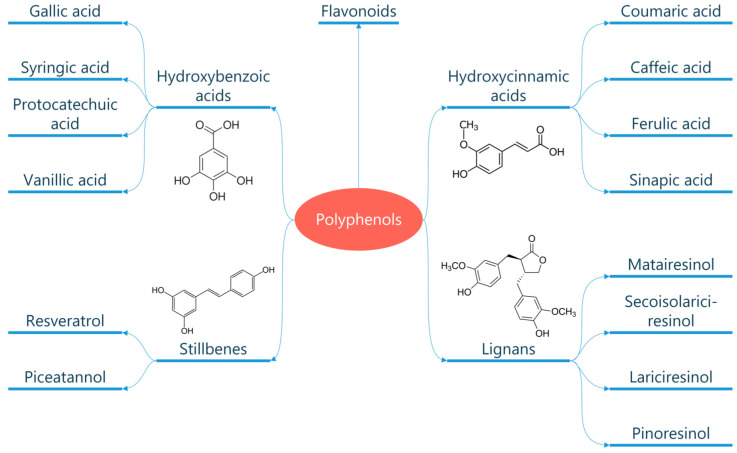
Classification of polyphenols. Polyphenols are natural compounds found in plant-based foods and beverages. They can be classified into different subclasses based on their chemical structure, including phenolic acids, flavonoids, stilbenes, and lignans.

**Figure 3:**
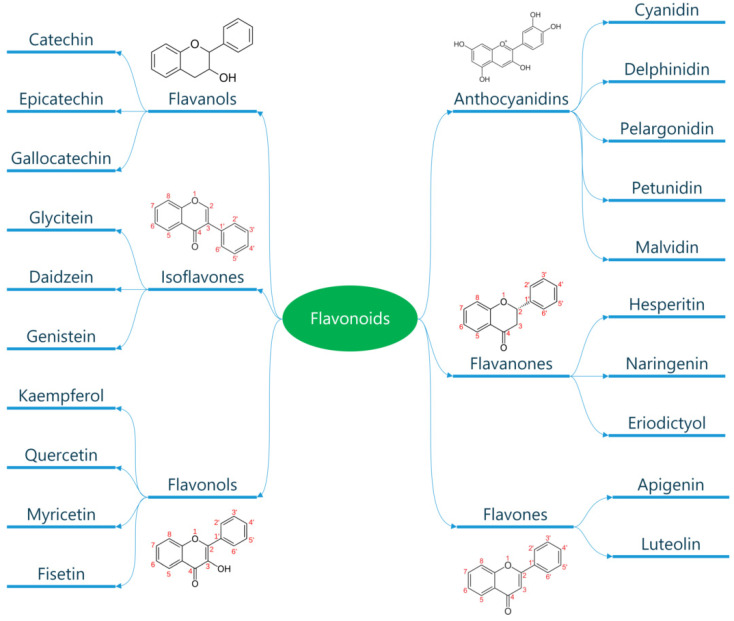
Classification of flavonoids. Flavonoids are a subclass of polyphenols and can be classified into flavonols, flavones, isoflavones, flavanones, antho-cyanidins, and flavanols based on their ring structure. Flavonoids have diverse biological activities and potential health benefits, including antioxidant and anti-inflammatory effects.

Several thousand molecules with a polyphenol structure (i.e., several hydroxyl groups on aromatic rings) have been identified in higher plants, and several hundred are found in edible plants. These molecules are secondary metabolites of plants and are generally involved in defending against ultraviolet radiation or aggression by pathogens. These compounds may be classified into different groups as a function of the number of phenol rings that they contain, and of the structural elements that bind these rings to one another. Distinctions are thus made between phenolic acids, flavonoids, stilbenes, and lignans (Figure 2) [147]. The flavonoids, which share a common structure consisting of two aromatic rings (A and B) that are bound together by three carbon atoms that form an oxygenated heterocycle (ring C), may themselves be divided into six subclasses as a function of the type of heterocycle involved: flavonols, flavones, isoflavones, flavanones, antho-cyanidins, and flavanols (catechins and proanthocyanidins) (Figure 3) [148]. In addition to this diversity, polyphenols may be associated with various carbohydrates and organic acids, and with one another.

More than 8000 phenolic structures are currently known, and among them over 4000 flavonoids have been identified [149]. The richest sources are fruit and vegetables. These substances are found in high concentrations in red wine, berries, and dark-colored vegetables; in fact, it is the polyphenols that give the intense color to some fruit and vegetables [150]. Polyphenol content is affected by several variables. Environmental factors have a major effect on polyphenol content: exposure to sunlight, for example. In addition, although very few studies have directly addressed this issue, the polyphenol content of vegetables produced by organic or sustainable agriculture is certainly higher than that of vegetables grown without stress, such as those grown in conventional or hydroponic conditions. This was shown recently in strawberries, blackberries, and corn [151]. Storage may also affect the content of polyphenols, which are easily oxidized [150]. Methods of culinary preparation also have a marked effect on the polyphenol content of foods [152]. On the basis of these considerations, we believe that when it comes to obtaining nutrients, the diet—not supplements—should be the primary source. The consumption of a balanced, unprocessed diet full of high-quality, raw organic foods, especially fruits and vegetables, assures the acquisition of the essential nutrients and antioxidants the body requires to achieve and maintain optimal health.

### 6.1. What Are the Best Antioxidant-Rich Foods That Should Make Up the Diet?

#### 6.1.1. Fresh, Organic Vegetables

Most edible vegetables, especially the green leafy ones, are loaded with potent phytochemicals, which are plant compounds that act as antioxidants. These phytochemicals can help reduce inflammation and eliminate carcinogens, protecting the body from a variety of health threats. However, to maximize the antioxidants in vegetables, they must be consumed raw, in a state closest to when they were harvested. Indeed, different types of heat treatment, such as boiling, baking, frying, or microwaving, can reduce the total antioxidant capacity of foods, affecting their ability to prevent lipid peroxidation [153,154]. Juicing is highly recommended so as to absorb all the nutrients in the vegetables—it is one of the healthiest antioxidant drinks that can be added to the diet. The pulp can also be eaten instead of throwing it away. Sprouts are also powerful sources of antioxidants, minerals, vitamins, and enzymes that promote optimal health. In particular, those of broccoli and red cabbage have been found to contain much more vitamin C and other radical scavenging activities than mature vegetables, and they also appear to be more palatable to young people [155].

Recommendations: Overall, incorporating fresh, organic vegetables and sprouts into the diet can help boost antioxidant intake, reduce inflammation, and promote optimal health. Choosing the right preparation methods can also help maximize the overall antioxidant content of food, ensuring that the full range of benefits provided by these healthy foods is obtained [150].

#### 6.1.2. Fruits

Fruits are a great source of nutrients, including vitamins, minerals, and fiber. In addition, many fruits contain phytochemicals, which are plant-based compounds that can provide health benefits. Fresh berries such as blueberries, blackberries, cranberries, and raspberries are the best antioxidant fruits, as they contain powerful phytochemicals that directly inhibit the DNA binding of certain carcinogens [156]. For example, anthocyanins, which are a type of flavonoid found in many berries, have been shown to inhibit the growth of cancer cells in laboratory studies [157]. Other phytochemicals found in berries, such as ellagic acid and quercetin, have also been shown to have anticancer effects [158,159]. Berries are also great sources of antioxidants such as vitamin C, carotenes, and carotenoids, as well as nutrients such as zinc, potassium, iron, calcium, and magnesium. Moreover, research has shown that daily consumption of antioxidant-rich fruits, such as berries, may help to improve various markers of cardiovascular health, including blood pressure, cholesterol levels, and endothelial function, leading to a significantly reduced risk of coronary heart disease [160,161,162]. Other antioxidant-rich fruits include citrus fruits such as oranges and lemons, which are high in vitamin C and flavonoids [163]. Apples, especially unpeeled, are also rich in antioxidants such as quercetin, catechin, and chlorogenic acid [164]. Grapes, especially red and purple varieties, are also high in antioxidants, including resveratrol [165]. In addition, fruits rich in potassium, such as bananas, cantaloupe, and avocados, have been associated with a reduced risk of cardiovascular disease [166]. Potassium helps to regulate blood pressure by counteracting the effects of sodium on the body.

Recommendations: To increase your intake of antioxidants and phytochemicals, we recommend that you consume fresh berries regularly, as they have been shown to have anticancer and cardiovascular benefits. We also recommend that you include other antioxidant-rich fruits in your diet, such as citrus fruits, apples, grapes, and bananas, as they can also provide you with health benefits. However, we advise that you should consume fruits in moderation, as they contain fructose, high amounts of which can be detrimental to health; this is true especially if too much fruit is consumed at dinner, since it strongly stimulates insulin production, especially in overweight people with insulin peripheral resistance or who are diabetic [167].

#### 6.1.3. Nuts

Pecans, walnuts, and hazelnuts are excellent antioxidant foods that can boost your heart health and overall health [168,169]. Nuts are known to contain high levels of monounsaturated and polyunsaturated fats, fiber, minerals, vitamins, and various bioactive compounds that offer numerous health benefits. Research has shown that incorporating nuts into your diet may help lower the risk of coronary artery disease and hypertension. The possible ways in which nuts can help prevent these conditions include improving the lipid profile of the blood, reducing insulin resistance, and modulating inflammation, oxidative stress, and endothelial function [170,171].

Recommendations: Look for nuts that are organic and raw, not irradiated or pasteurized. Peanuts are usually less than ideal, as they are usually pesticide-laden and can be contaminated with a carcinogenic mold called aflatoxin [172].

#### 6.1.4. Herbs and Spices

Aside from being an abundant source of antioxidants, these can have potential anti-cancer benefits. Herbs and spices differ mainly by source, as herbs typically come from the plant’s leaves while spices come from the bark, stem, and seeds. Both have been used for thousands of years to flavor foods and treat illnesses. Some of your best choices are ground cloves, ground cinnamon, oregano, turmeric, ginger, and garlic. For example, curcumin, the active ingredient in turmeric, has been shown to improve endothelial function and decrease inflammation, both of which are important in reducing the risk of cardiovascular disease and coronary artery disease. Studies have shown that curcumin can increase the activity of antioxidant enzymes, while also decreasing the levels of various oxidative stress markers and ROS [173]. In addition to its antioxidant properties, curcumin has been found to have potent anti-inflammatory effects, which are also important for protecting the cardiovascular system. Chronic inflammation is a major risk factor for cardiovascular disease, and curcumin has been shown to inhibit the production of pro-inflammatory cytokines and other mediators of inflammation, such as NF-κB [174]. Studies have also shown that curcumin can improve lipid profiles by reducing levels of total cholesterol, LDL cholesterol, and triglycerides, while increasing levels of HDL cholesterol [175]. In addition, curcumin has been found to have antithrombotic effects, which may help prevent the formation of blood clots and reduce the risk of heart attacks and strokes [176]. Similarly, research has suggested that ginger, another commonly used herb, may have cardioprotective properties, because it may help lower blood pressure, reduce inflammation, and improve lipid metabolism, all of which are important factors in preventing cardiovascular disease [177].

Recommendations: Ideally, you should only opt for fresh herbs and spices, as they are healthier and have higher antioxidant levels than processed, powdered versions. For example, the antioxidant activity of fresh garlic is 1.5 times higher than that of dry garlic powder [178]. Moreover, adding fresh herbs and spices to your meals not only boosts their flavor and nutrition but can also help you reduce your intake of unhealthy additives. Processed and pre-packaged foods often contain high amounts of salt, sugar, and unhealthy fats to enhance their flavor, which can be detrimental to your health in the long run. By using fresh herbs and spices, you can avoid these additives and enjoy the natural flavors of your food. Finally, using herbs and spices to flavor your food can help you reduce your sodium intake, which is crucial for individuals with high blood pressure.

#### 6.1.5. Organic Green Tea

This antioxidant-rich drink contains epigallocatechin-3-gallate (EGCG), a catechin polyphenol and one of the most powerful antioxidants known today. EGCG benefits you by lowering your risk of heart attack and stroke, glaucoma, high cholesterol, and more. Studies have also found that it can improve your exercise performance, increase fat oxidation, and even help prevent obesity due to its regulatory effect on fat metabolism [179,180,181]. However, remember that not all green teas are created equal. Some processed green tea brands can contain very little or no EGCG at all. Some tea bags are also contaminated with fluoride or contain hazardous plastics that can leach into your tea when brewing.

Recommendations: To ensure you are drinking high-quality green tea, we advise buying only organic, loose-leaf tea from a reputable source. In addition, tea is not recommended for people that suffer from some forms of cardiac arrhythmias, as its alkaloid content can worsen such a problem, even if low-dose green tea intake has been related to a reduced incidence of both paroxysmal and persistent atrial fibrillation [182].

The importance of oxidative stress on endothelial function as a trigger for vessel damage and cardiovascular events has been established. In experimental animal models of atherosclerosis, hypercholesterolemia, hypertension, and diabetes, associations between oxidative stress and impaired endothelial function have been demonstrated. Among many biological changes that occur in the vessel wall under these conditions, reduced bioavailability of nitric oxide (NO) in a setting of increased superoxide anion levels seems to be a uniform underlying abnormality. Recent studies extended this potential mechanism to patients with coronary artery disease by demonstrating increased superoxide production of human blood vessels in association with endothelial vasomotor dysfunction and with clinical risk factors [183,184]. Furthermore, endothelial dysfunction in patients with coronary artery disease or coronary risk factors could be reversed by the administration of agents capable of scavenging superoxide, such as vitamin C [185,186]. These findings suggest that increased oxidative stress may be an important mechanism for impaired endothelial function in patients with atherosclerosis or cardiovascular risk factors.

Nowadays, there is growing interest in the role of dietary polyphenols in the prevention and treatment of heart diseases. Polyphenols have been shown to have a range of beneficial effects on cardiovascular health, including improving endothelial function, reducing inflammation, and lowering blood pressure. Several epidemiological studies have reported that high intake of dietary polyphenols is associated with a reduced risk of heart diseases, leading to a lower risk of coronary heart disease and a lower incidence of heart failure [187,188]. Some studies have reported significant improvements in cardiovascular risk factors, such as blood pressure, cholesterol levels, and endothelial function, with supplementation of polyphenols [189,190,191]. One of the most well-known examples of the potential health benefits of polyphenols is the French paradox, whereby moderate red wine consumption in a diet otherwise high in saturated fats is associated with a lower risk of cardiovascular mortality in French people from the Bordeaux region [192]. Polyphenols can provide anti-fibrotic and myocardial protection by inhibiting oxidative stress and molecular pathways involved in heart fibrosis, and they can also promote vasodilation by increasing NO release, which improves vascular function by relaxing smooth muscle, inhibiting platelet aggregation, and increasing prostacyclin production. Moreover, polyphenols have been shown to have anti-diabetic effects by reducing blood glucose and glycated hemoglobin A1c levels, to be able to modulate liver function and lipid metabolism and to be effective in combating obesity. In fact, supplementation of polyphenols from red grapes leads down an anti-inflammatory pathway, causing weight reduction in obese individuals [193].

**Figure 4:**
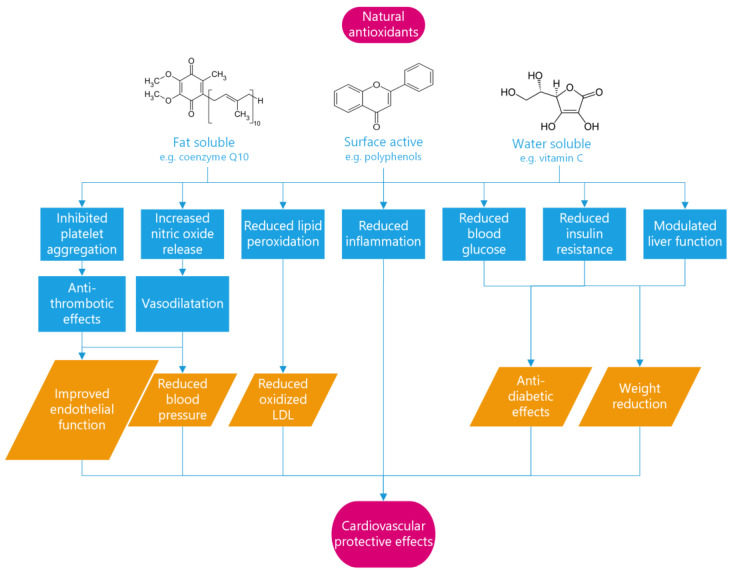
Mechanisms of antioxidant protection in cardiovascular diseases. Antioxidants act through multiple pathways to improve endothelial function, reduce blood pressure, prevent the formation of oxidized low-density lipoprotein (LDL), and provide anti-diabetic and weight-reducing effects. These mechanisms counteract the effects of free radicals, which have been implicated in the pathology of coronary diseases, and reduce the risk of cardiovascular events.

Overall, the evidence suggests that polyphenols have a beneficial impact on heart diseases (Figure 4).

### 6.2. Are Nutritional Supplements as Effective and Safe as Natural Food Sources?

Nutritional supplements are increasingly popular in the healthcare industry as people seek to augment their diets with vitamins, minerals, and other compounds thought to promote wellness and combat disease. Although these products can be effective in certain situations, they are often misused, overhyped, and even harmful to human health. While it is true that some dietary supplements can help meet nutrient needs, research suggests that they are often less effective than natural foods. Several studies suggest that dietary vitamin C is more protective than supplements and is associated with a reduced incidence of chronic diseases, including stroke, coronary heart disease, and various types of cancer [194]. Similarly, recent studies found that supplementing with vitamin E did not lower the risk of heart disease, whereas consuming vitamin E through foods such as nuts and seeds did [195,196]. Moreover, excessive intake of vitamin supplements can have harmful effects on the body, particularly when taken in high doses over long periods of time. For example, high doses of vitamin A can cause liver damage, while excessive intake of vitamin D can lead to hypercalcemia, a condition characterized by high levels of calcium in the blood. Therefore, even commonly used supplements, such as multivitamins, vitamin E, and folic acid, appear to have limited or no benefit, and some may be harmful [197].

The bioavailability of nutrients from whole foods is generally higher than that of supplements, and they also contain other compounds such as fiber, antioxidants, and phytochemicals that may have synergistic effects on health. There is also a concern that taking supplements may lead to a false sense of security and encourage unhealthy dietary practices. For example, some individuals may take supplements as a means of compensating for a poor diet, rather than making healthy dietary choices. Additionally, it is important to note that the dietary supplement industry is largely unregulated, and many products may not contain the ingredients listed on the label, or may be contaminated with harmful substances. For these reasons, it is generally best to obtain nutrients from whole foods rather than supplements.

## 7. Future Perspectives

The complex interplay between oxidative stress, inflammation, and cardiovascular diseases poses several challenges and opportunities for future research. On the one hand, there is a need to better understand the molecular mechanisms underlying the pro-oxidant and anti-oxidant effects of different dietary polyphenols and their metabolites in the context of obesity and coronary artery disease. On the other hand, there is a potential to develop novel therapeutic strategies based on the modulation of oxidative stress and inflammation by natural antioxidants. Firstly, future studies should investigate the impact of lifestyle interventions on oxidative stress and inflammation in coronary artery disease. There is growing evidence that lifestyle interventions, such as dietary modifications, exercise, and stress reduction techniques, can have a significant impact on reducing oxidative stress and inflammation in patients with coronary artery disease [198,199]. Thus, it would be important to examine the effectiveness of such interventions and determine the optimal strategies for their implementation. Secondly, further research is needed to identify novel biomarkers of oxidative stress and inflammation that can independently predict cardiovascular events. Although several biomarkers have been proposed in the literature, their predictive value remains uncertain [200,201,202]. Therefore, it is crucial to identify reliable biomarkers that can help clinicians to assess the risk of coronary artery disease and monitor disease progression. However, we believe that assessing the by-products of free radicals can have a significant clinical impact when combined with endothelial functional tests, as previously discussed in this paper. Thirdly, investigations should focus on the development of new therapeutic strategies that can target oxidative stress and inflammation in coronary artery disease. Although several antioxidants and anti-inflammatory agents have been proposed as potential therapies for coronary artery disease, their efficacy and safety are still uncertain [203,204]. Therefore, it is important to conduct well-designed clinical trials to determine the optimal dose, duration, and safety of such therapies. Finally, a number of questions that deserve further investigation remain open:

- What are the optimal doses and combinations of dietary polyphenols to achieve maximal protection against oxidative stress and inflammation in patients with coronary artery disease?
- How do genetic and environmental factors influence the bioavailability, metabolism, and activity of dietary polyphenols and their metabolites in different tissues and organs?
- How can oxidative stress biomarkers be improved to reliably reflect oxidative status and the risk of cardiovascular events in these patients?
- What are the long-term effects and safety of antioxidant supplementation on cardiovascular outcomes and mortality in obese patients with coronary artery disease?

Answering these questions may provide new insights into the role of oxidative stress in obesity-related cardiovascular diseases and pave the way for the development of personalized and effective interventions based on dietary polyphenols or their derivatives.

## 8. Conclusions

The rampant diffusion of factors generating free radicals is a real threat for the health of the endothelium and the coronaries, and it is evident that the engine that has generated such acceleration of free radicals’ formation is man-made. A definite change in direction is rapidly needed. In the meantime, coronary patients should avoid all those factors that can generate free radicals, putting the endothelium under siege; reduce weight to tackle obesity and chronic inflammation that, along with free radicals, create the perfect storm for atherosclerosis generation; and finally optimize the intake of natural and organic food with a high content of balanced and protecting antioxidants.
